# Speech therapy program for the rehabilitation of smell

**DOI:** 10.1590/2317-1782/e20250086en

**Published:** 2026-03-27

**Authors:** Carla Patrícia Hernandez Alves Ribeiro César, Keyllam Machado de Lima, Otávio Messias Silva de Oliveira, Lorena Mikaelly Nascimento Santos, Vanessa Silva Dantas, Danielle Ramos Domenis, Rafael Ciro Marques Cavalcante, Raphaela Barroso Guedes-Granzotti, Kelly da Silva

**Affiliations:** 1 Departamento de Fonoaudiologia, Universidade Federal de Sergipe – UFS - São Cristóvão (SE), Brasil.; 2 Programa Associado de Pós-graduação em Fonoaudiologia, Universidade Federal da Paraíba, Universidade Federal do Rio Grande do Norte e Universidade Estadual de Ciências da Saúde de Alagoas, Brasil.; 3 Departamento de Fonoaudiologia, Universidade Federal de Sergipe – UFS - Lagarto (SE), Brasil.; 4 Departamento de Farmácia, Universidade Federal de Sergipe – UFS - Lagarto (SE), Brasil.

**Keywords:** Olfaction Disorders, Olfactory Training, Smell, Rehabilitation, Speech Therapy

## Abstract

**Purpose:**

To evaluate the effectiveness of a speech-language pathology-based therapeutic intervention for olfactory dysfunctions.

**Methods:**

The speech-language olfactory rehabilitation program was applied to eight patients with complaints of reduced olfaction due to persistent post-viral infection (1 to 2 years post-infection) and confirmed hyposmia, as determined by the Connecticut Chemosensory Clinical Research Center test. The intervention consisted of 13 in-person sessions of 45 minutes each (the first and last for assessment), which included strategies aimed at increasing awareness of olfaction; nasal breathing and the need for frequent hygiene (nasal and intraoral); perception, identification, and contextualization of odor stimuli using four primary odors; the Nasal Airflow-Inducing Maneuver; as well as taste association and home-based training using the MedSmell® olfactory training kit, three times per day. The project was previously approved by the ethics committee and registered in the Brazilian Registry of Clinical Trials.

**Results:**

The patients had a mean age of 53.29 ± 21.92 years, with an even distribution between the sexes. All patients showed improvement in olfactory function: six (75%) recovered to normosmia, one (12.5%) improved from anosmia to moderate hyposmia and another (12.5%) from anosmia to mild hyposmia. The olfactory training kit was used daily at home, and all patients completed the program, reporting perceived therapeutic benefits.

**Conclusion:**

The speech-language pathology intervention was effective in the rehabilitation of olfactory disorders of viral origin, though further studies with larger samples are needed to generalize the results.

## INTRODUCTION

The sense of smell is one of the most important and ancient functions in human evolution, as it enables the perception of diverse odor stimuli through sensory receptors located in the nasal cavity. These receptors establish direct synaptic connections with the olfactory bulb at the base of the brain, which in turn projects to multiple cortical regions, as well as with the limbic system, thereby mediating the affective components of odors, such as pleasure and aversion^(1)^.

Dysfunctions in this sensory perception significantly impact a person's life and can result from various etiological factors, including viral infections, traumatic brain injuries, smoking habits, neurodegenerative and systemic diseases, as well as iatrogenic causes. Olfactory dysfunctions have gained prominence since the COVID-19 pandemic, as they represent a salient symptom in infected patients and may persist for up to three years post-infection^(2)^.

The diagnostic pillars of olfactory dysfunctions consist of the clinical history and the application of standardized, validated psychophysical tests, which verify everything from the absence of smell (anosmia) to partial reduction (hyposmia). Therefore, the most widely used and internationally recognized tests are the University of Pennsylvania Smell Identification Test (UPSIT)^(3)^, validated for the Portuguese language^(4)^; the Connecticut test developed by American researchers^(5)^ in 1988 and subsequently adapted for Brazil^(6)^; and the Sniffin’ Sticks^(7)^, wich have already been transculturally adapted for the Brazilian pediatric population^(8)^, among others.

Therapeutic interventions are guided by their etiological factors, with olfactory training representing one possible treatment option. The lower the degree of dysfunction and the younger the patient, the better the prognosis, as well as the possibility of addressing the etiological factor, although there may be therapeutic limitations in this regard^(9)^.

Speech-language pathology specialties that may contribute to the functional treatment of olfaction are Orofacial Myofunctional Therapy and Dysphagia, as these areas directly address the orofacial and cervical regions. Moreover, olfactory and gustatory dysfunctions can compromise the quality of life of affected individuals^(10)^.

The current research is justified because, to date, no scientific publications on standardized speech-language therapy interventions for patients with hyposmia of different etiological origins. These include viral infections (especially SARS-CoV-2 since 2020), traumatic brain injuries, neurodegenerative diseases such as Alzheimer's and Parkinson's, natural aging, and consequences of radiation and/or chemotherapy treatment for craniocervical cancer, among others.

Given the above, this research aimed to evaluate the effectiveness of speech-language therapy interventions for olfactory dysfunctions in patients with post-COVID-19 syndrome, through a pre- and post-intervention comparison of olfactory performance using a nationally and internationally recognized test.

## METHODS

This is a randomized, prospective, before-and-after, triple-blind clinical trial, approved in accordance with the Research Ethics Committee (CAAE No. 61448422.7.0000.0217; Approval No. 5.658.792), and registered in the Brazilian Registry of Clinical Trials (ReBEC) under number RBR-23k6dgq.

To compose the sample, a survey of all medical records of patients hospitalized with COVID-19 between March 11, 2020, and May 22, 2022, were reviewed at two hospitals that served as referral centers for these cases in the state of Sergipe. In December 2023, patients were contacted by telephone and invited to participate in the research. For those who accepted, appointments were scheduled to sign the Informed Consent Form, undergo an interview, and an olfactory assessment on pre-established dates between January and February 2024. Randomization was applied, of the 49 patients evaluated, 18 exhibited persistent olfactory dysfunction between one and two years post-infection, with 13 confirming hyposmia. These thirteen were subsequently invited to participate in the Speech Therapy Olfactory Rehabilitation Program (PROL).

It is important to note that a prior sample size calculation was performed, considering a large effect size (d = 0.8), a significance level of 5% (α = 0.05), and a statistical power of 80%. The minimum number of participants required to detect a significant difference between pre- and post-intervention measurements was 13. However, due to eligibility criteria and participant refusal, the study was initiated with nine patients and concluded with eight, as presented in the results section.

Inclusion criteria included individuals aged 18 to 60 years who presented with severe COVID-19, who were hospitalized for treatment of the respiratory viral infection, and consented to participate in speech therapy. Exclusion criteria encompassed individuals with cognitive impairments; those who tested positive for COVID-19 at the time of assessment; those with difficulties in orally responding to questionnaires and complying with therapeutic guidelines; and those using medications known to interfere with olfactory assessment and rehabilitation, such as carboplatin, cisplatin, cyclophosphamide, doxorubicin, fluorouracil, methotrexate, levamisole, and vincristine, whether due to mucosal inflammation/irritation or neurotoxicity. Patients reporting olfactory alterations but obtained normal results were also excluded due to possible parosmia, subtle or intermittent dysfunction, hyperosmia, odor-specific alterations, isolated retronasal olfactory dysfunction, among other factors. To apply these criteria, patients underwent an interview on the day of data collection, before the formal evaluation.

To minimize the risk of bias^(11)^, the following measures were implemented: (1) patient allocation was determined by the examiner; specifically, examiners did not administer therapy and therapists did not conduct assessments. To this end, one of the project coordinators was the only person with access to the assessments and therapies results, and a central research results center was established; (2) containers used in the olfactory test were standardized and uniform so that patients could not identify the test stimuli; (3) patients, outcome assessors, and therapists were blinded (triple-blind design), since the completed examination protocols, after being performed by the assessors, were placed in sealed and numbered brown envelopes by the central results center, thereby preventing assessors and therapists from accessing the information; (4) all procedures adopted were identical among patients^(12)^.

Regarding the procedures, prior to both the initial olfactory assessment and the subsequent reassessment, a COVID-19 antigen test r was administered. Results were reported as detected (positive) or not detected (negative). Participants who tested negative proceeded to the next stage of the protocol.

For the evaluation of olfactory function, the Connecticut Chemosensory Clinical Research Center (CCCRC) test, marketed by MedSmell® and standardized for use in Brazil, was employed^(6)^. The research kit comprised: (a) eight vials containing solutions of n-butyl alcohol (butanol) in different concentrations (4%, 1%, 0.4%, 0.1%, 0.05%, 0.01%, 0.005%) and one vial with distilled water (control), used for olfactory threshold assessment; (b) eight vials containing odoriferous substances, including coffee powder, cinnamon powder, talcum powder, peanut brittle, chocolate powder, neutral soap, naphthalene, for odor identification; (c) one vial containing menthol, for the evaluation of trigeminal nerve afferents; (d) an eye mask; (e) an instruction manual; and (f) response sheets/tables for recording results.

The analysis comprised both quantitative and qualitative components: quantitative evaluation was based on the determination of the olfactory threshold, whereas qualitative evaluation involved recognition and naming of the odor stimuli. Each nostrils was tested separately, and patients were blindfolded to keep their eyes closed. The result was the sum of the scores, obtaining the arithmetic mean:


$$\frac{\begin{array}{c} right\;nostril\;threshold+left\;nostril\;threshold+\\ right\;nostril\;odor\;recognition+\\ left\;nostril\;odor\;recognition \end{array}}{4}$$

Based on the mean score, olfactory function was classified as follows: nomosmia (score between 6.0-7.0 points), hyposmia (score between 5.0–2.0 points, mild between 5.75–5.0 points, moderate between 4.75–4.0 points, and severe between 3.75–2.0 points), and anosmia (score between 1.75 and zero points).

The PROL protocol consisted of 13 weekly in-person sessions, each lasting 45 minutes, with the first and thirteenth sessions dedicated to baseline evaluation and re-evaluation, respectively. The general objective was to improve olfactory function, and its specific objectives were: 1) Promoting nasal breathing; 2) Detecting, discriminating, naming, and memorizing odors (always preceded by nasal hygiene, use of a bandage during the session, and performance of the Nasal Airflow Inducing Maneuver - which is the “polite yawn technique”, involving deep nasal inspiration and expiration with mandibular lowering without lips opening, generating negative intraoral and oropharyngeal pressure, thus stimulating retronasal olfaction^(13)^, taste, and affective memory related to smell, all used together for this purpose); and 3) Providing guidance on home-based activities and emphasizing the importance of olfaction for nutrition. Participants received the MedSmell® kit for daily olfactory training at home, along with instructions for its use. This intervention was supported by research funding from National Council for Scientific and Technological Development of Brazil (CNPq).

Home olfactory training and hygiene were performed daily (at least three times per day), and training records were kept on a form. It should be noted that the PROL was described using the following structure: International Statistical Classification of Diseases and Related Health Problems (ICD-11), main findings^(12,14-16)^, objectives (general and specific) with the respective speech therapy interventions: number of sessions, content, resources, and strategies (available in the Supplementary Material).

Patients with 15% absenteeism were excluded from the study. Furthermore, the selective outcome of interest was defined, classifying therapeutic success rates between 80-100% as satisfactory, moderate between 79-60%, fair between 59-40%, poor between 39-20%, and unsatisfactory between 19-0%.

## RESULTS

Of the eight patients enrolled, four were female and four were male, with a mean age of 55.50 ± 21.24 years.

The therapeutic objectives were pursued without modifications to the previously established strategies. Patients demonstrated cooperation and adherence throughout the sessions, describing progressive improvements in their clinical condition. Comparative results of the pre- and post-therapy assessment, along with the respective conduct are presented in Chart 1.

**Chart 1:** Results before and after olfactory therapy, including comparative outcomes and corresponding actions

**Subjects**	**Pre-therapy results (Average score and classification)**	**Post-therapy results (Average score and classification)**	**Comparative considerations and clinical conduct**	**Therapeutic outcome**
1	3.5 points Severe Hyposmia	7.0 points Normosmia	Olfactory function improved and fully restored. Clinical outcome: discharge from speech-language therapy.	Satisfactory (100%)
2	4.0 points Moderate Hyposmia	6.0 points Normosmia	Olfactory function improved and fully restored. Clinical outcome: discharge from speech-language therapy.	Satisfactory (85.71%)
3	1.5 points Anosmia	4.0 points Moderate Hyposmia	Olfactory function improved. but the sense of smell was not restored. Clinical outcome: continue therapy.	Fair (57.14%)
4	1.5 points Anosmia	5.25 points Mild Hyposmia	Olfactory function improved, but the sense of smell was not restored. Clinical outcome: continue therapy.	Moderate (75%)
5	3.25 points Severe Hyposmia	6.0 points Normosmia	Olfactory function improved and fully restored. Clinical outcome: discharge from speech-language therapy.	Satisfactory (85.71%)
6	3.75 points Severe Hyposmia	7.0 points Normosmia	Olfactory function improved and fully restored. Clinical outcome: discharge from speech-language therapy.	Satisfactory (100%)
7	2.75 points Severe Hyposmia	7.0 points Normosmia	Olfactory function improved and fully restored. Clinical outcome: discharge from speech-language therapy.	Satisfactory (100%)
8	4.5 points Moderate Hyposmia	7.0 points Normosmia	Olfactory function improved and fully restored. Clinical outcome: discharge from speech-language therapy.	Satisfactory (100%)

**Source:** Authors’ elaboration.

## DISCUSSION

The aim of this study was to evaluate the effectiveness of speech-language therapy interventions targeting olfactory dysfunctions in patients with post-COVID-19 syndrome, given that such alterations negatively impact the mental health and quality of life of affected individuals^(17)^. Many patients infected with the SARS-CoV-2 virus presented acute episodes of olfactory loss, but with recovery of function. However, in approximately 5% to 10% of cases with anosmia, the condition persisted^(18)^. The literature has referred to such manifestations as long COVID, defined by the presence of continuous or post-acute long-term multisystemic sequelae after viral recovery. For the early detection of long COVID, routine ancillary assessments (including imaging and laboratory examinations) are currently indispensable, particularly olfactory tests^(19)^.

According to the literature, the most frequently employed therapeutic approaches for olfactory dysfunctions include topical pharmacological agents, such as corticosteroids^(12)^, dietary supplements containing palmitoylethanolamide and luteolin^(20)^, those involving olfactory stimulus training, combined strategies (medication and olfactory training^(20,21)^), and the application of low-frequency photobiomodulation^(22)^. In the present study, to specifically assess the impact of olfactory training, patients using medication were excluded from the sample to avoid potential bias in the analysis. Photobiomodulation was not implemented due to the requirement for high-cost equipment and specialized professionals trained in its application.

Among the strategies employed in PROL, the combined use of nasal irrigation with saline solution, retronasal olfaction, nasal bandaging (to promote nostril dilation during face-to-face sessions), and concomitant interventions involving gustatory stimulation and affective memories associated with olfactions yielded positive results in this study.

Regarding nasal hygiene, this outcome can be explained by the fact that a clean nasal cavity allows for greater airflow efficiency and, consequently, facilitates both nasal breathing and clearance. Moreover, it enhances mucociliary clearance function, as it promotes the elimination of viscous mucus, reduces inflammatory mediators, and increases the frequency of ciliary beating, although the precise mechanisms underlying these effects remain unclear^(22,23)^.

It is also noteworthy that the Nasal Airflow-Inducing Maneuver (NAIM) is employed in totally laryngectomized patients to retroflexively stimulate olfactory function, thereby enabling odor perception and restoring the sense of smell, irrespective of the time elapsed since laryngectomy^(24)^. In the present study, this technique proved to be an effective complementary strategy, even in patients without laryngectomy.

The program's main strategy was to offer distinct primary odoriferous stimuli (floral, resinous, aromatic, and fruity), as recommended by a systematic review with meta-analysis^(25)^. This study demonstrated that olfactory training in patients infected with the SARS-CoV-2 virus produced satisfactory outcomes in general, with effects being more evident in cases presenting an acute clinical profile.

It is also noteworthy that, according to the literature, different odor categories and therapeutic durations have been employed, with the basis of the training based on Henning's "odor prism". In this model, patients are instructed to perform home-based training using a standardized set of four odors (phenylethyl alcohol (rose), eucalyptol (eucalyptus); citronellal (lemon); eugenol (clove) twice a day for 12 weeks^2^. In the present study, daily olfactory training at home was conducted with standardized odor stimuli from MedSmell® , complemented by eleven therapy sessions utilizing stimuli from the four primary odor categories, thereby achieving success in the proposed approach. The results obtained in this study corroborated the findings reported in the literature. However, in addition to home-based training, in-person follow-up was conducted, integrating olfactory and gustatory stimulation while revisiting patients’ previous experiences with the stimuli addressed during the sessions. This occurred because the sense of smell is intimately linked to the limbic system. Accordingly, whenever olfactory stimuli were employed in the sessions, efforts were made to recreate meaningful contexts and to verbally explore patients’ affective experiences with the presented odors, thereby yielding satisfactory outcomes in this regard.

The rationale for this improvement, according to another systematic review that discusses the olfactory nerve regeneration process^(26)^, is that such regeneration is possible through several mechanisms. These include the renewal of basal cells (both horizontal and globular) located in the olfactory epithelium, which serve as precursors cells of olfactory receptor neurons; by increasing the enzymatic activity of guanine nucleotide-binding proteins G(olf) alpha subunit (GNAL) and adenylyl cyclase (ADCY)-3f, as well as increased expression of glial fibrillary acidic protein (GFAP) and migrating of neuroblasts. Thus, the biochemical and cellular changes in the nasal epithelium appear to facilitate an increase in the olfactory bulb and, consequently, in several central regions involved in olfactory processing, justifying the reason for the success achieved through olfactory training. Therefore, it is hypothesized that repeated stimulation with odoriferous stimuli promoted neurogenesis of the olfactory bulb and strengthened the cortical pathways associated with olfaction.

Further studies involving olfactory training supported by techniques imaging exams (computed tomography and magnetic resonance imaging) are required to corroborate and deepen the understanding of the neuroplasticity involved. This need is exemplified by the study conducted by researchers^(27)^ with patients presenting post-traumatic dysfunction, developing a prognostic score composed of five items, achieving a sensitivity of 76.5% and a specificity of 97.1% in predicting whether individuals would respond favorably to olfactory training.

The results of this study indicate relevant implications for clinical practice in speech-language pathology, especially in the context of rehabilitation virus-related olfactory dysfunctions. The significant improvement in patients’ sense of smell, with particular emphasis on the recovery of normal olfactory function in 75% of cases, reinforces the therapeutic potential of the speech-language pathology strategies applied, despite the relatively small sample size. These findings highlight the importance of olfactory rehabilitation as part of clinical approaches in post-infectious contexts, expanding the scope of speech-language pathology practice and potentially proving useful in cases with other etiological factors associated with anosmia or hyposmia. From a research perspective, the need for studies with larger samples and controlled designs is evident to validate and consolidate the effectiveness of these interventions. From a public policy standpoint, the inclusion of structured olfactory rehabilitation programs in health services may contribute to improving the quality of life of patients with viral sequelae, optimizing resources, and promoting more comprehensive care for the sensory health of individuals affected by the condition investigated in this study.

Among the limitations of this study, the small sample size can be noted, which prevents generalization of the findings. Nevertheless, the benefits observed and reported by the patients justified the study’s execution and the dissemination of its results.

For future directions, it is suggested to compare the results with patients presenting other clinical conditions that account for olfactory dysfunction, as well as to compare exclusive home-based olfactory training with combined training (therapy plus home practice), in order to determine whether differences exist between these modalities. Another possibility would be the use of virtual reality associated with the delivery of odoriferous stimuli, thereby enhancing participants’ experience of the presented situation.

## CONCLUSION

Based on the speech-language pathology rehabilitation program for olfactory function, conducted as a triple-blind clinical trial, it can be concluded that the program, designed for patients with viral dysfunction, produced satisfactory effects in most of the samples studied. Nevertheless, the results should be interpreted with caution due to the sample size.

## Material Suplementar

Este artigo acompanha material suplementar.

Programa Fonoaudiológico de Reabilitação do Olfato (PROL)

Este material está disponível como parte da versão online do artigo na página https://doi.org/10.1590/2317-1782/e20250086pt
