# Supplementary material for: Speech therapy program for the rehabilitation of smell
Source: Codas. 2026 Mar 27;38(2):e20250086. doi: 10.1590/2317-1782/e20250086en (PMC13075829; doi:10.1590/2317-1782/e20250086en)
Supplement: Programa Fonoaudiológico de Reabilitação do Olfato (PROL) [file codas-38-2-e20250086-suppl01.pdf]

Supplementary Material

Speech Therapy Olfactory Rehabilitation Program (PROL)

I. International Statistical Classification of Diseases and Related Health Problems (ICD-11)

R43 Smell and taste disorders.

R43.0 Anosmia

R43.1 Parosmia

R43.2 Parageusia

R43.8 Other smell and taste disorders and those not specified

II. Key findings

1) Incomplete and delayed recovery from sudden olfactory loss secondary to viral infections, specifically COVID-19[13].

2) Altered sense of taste (dysgeusia), altered sense of smell (hyposmia or anosmia), xerostomia, and hyposalivation after treatment for COVID-19[12, 14].

3) Increased risk of distorted odor perception (parosmia or troposmia) among young individuals affected by viral infection (in this case: COVID-19), with adverse impacts on quality of life[15].

III. General Objective

1) To rehabilitate olfactory function.

IV. Specific Objectives and Respective Therapeutic Interventions.

1) Conduct a patient interview and olfactory assessment.

Number of sessions: 1.

Resources: Anamnesis protocol, Connecticut test kit.

Strategies: Anamnesis with identification data, past history of the complaint, survey of possible etiological factors, previous and current treatments, impacts of hyposmia/anosmia on activities of daily living, and olfactory assessment.

2) To establish nasal breathing.

Number of sessions: 11.

Resources: Mirror, figures, blowing materials (Pró-Fono® breathing kit, party blower, and balloon), syringe for nasal cleaning with saline solution, and tongue scraper.

Strategy 1: Through video, illustration, and oral presentation by the therapist, the respiratory function will be explained, and nasal and tongue hygiene, which should be done daily (at least three times per day), will be demonstrated.

Strategies 2-11: Breathing techniques including nasal inspiration (I), pause (P), and nasal expiration (E), with a gradual increase in duration, combined with the prior application of an inverted "V" elastic bandage from the glabella to the nasal wing (applied at the beginning and removed at the end of the session by the therapist), provided there are no indications of allergy to the material. Blowing materials will be used to diversify activities within the therapeutic process.

3) To detect, discriminate, identify, and memorize odors.

Number of sessions: 11.

Resources: MedSmel® kit with four amber bottles containing watercolor paper discs impregnated with essences from distinct olfactory groups: lemon, rose, clove,e and eucalyptus (2 mL each), plus bottles with primary scents, as listed in the table below.

Chart. List of primary odors and stimuli employed weekly in-person therapies, indicating the session of use.

**Primary odors**	**Stimuli**	**Format – Session**
Floral	Talc	Johnson® MedSmell talc container - Session 2.
	Jasmine	Jasmine bar soap (Granado®) – Session 3.
	Lily	Phebo® brand bar soap (Lily) – Session 4.
	Rose	Nativa Spa bar soap, by Boticário® (Pink) – Sessions 5 and 6.
	Orange	Natura® brand bar soap, Ekos line (Orange and Orange Blossom) – Session 7.
	Lavender	Natura® brand bar soap, Ekos line (Lavender) – Section 8.
	Rose	Natural rose – Session 9.
	Chamomile	Box of tea (Leão® brand, chamomile flavor) – Session 10.
	Anise	Box of tea (Leão® brand, fennel flavor) – Session 11.
Resinous	Menthol	MedSmell® container – Sessions 2 and 10.
	Eucalyptus	Eucalyptus Oil (Granado® brand) – Sessions 3 and 7.
	Mint toothpaste	Close Up® brand toothpaste tube (Refreshing Mint flavor) – Sessions 4 and 11.
	Vanilla	Dr. Oetker® vanilla container – Sessions 5 and 9.
	Wood	Wood chips (obtained from carpentry shops) – Section 6.
	Plastic	Plastic bottle with water inside (left in the sun before use) – Session 8.
Aromatic	Clove	Small packet of whole cloves (Arisco® brand) – Section 2.
	Cinnamon	Small packet of cinnamon sticks (Arisco® brand) – Session 3.
	Lemongrass	Box of lemongrass tea (Leão® brand) – Session 4.

	Instant and ground coffee	Small container of instant and ground coffee (Pilão® brand) – Section 5.
	Chocolate bars and powder	Small container of Nescau powder (Nestlé®) – Session 6.
	Garlic	Jar of crushed garlic in brine (Arisco® brand) – Section 7.
	Onion	Fresh onion stem (actual product) – Session 8.
	Oregano	Packet of dried oregano (Kitano® brand) – Section 9.
	Paçoca (peanut candy)	Small packet of "Paçoquita" candies (Santa Helena® brand) – Section 10.
	Coriander	Bunch of fresh cilantro (real product) – Session 11.
	Cumin	Packet of ground cumin (Kitano® brand) – Section 11.
Fruity	Orange	Natural fruit and orange essence container (Arcolor® brand) – Session 2.
	Banana	Natural Fruit – Session 3.
	Passion fruit	Natural fruit and passion fruit essence container (Arcolor® brand) – Session 4.
	Pineapple	Cake and pineapple essence container (Arcolor® brand) – Session 5.
	Grapes and strawberries	Natural grapes and strawberry essence container (Arcolor® brand) – Session 6.
	Apple	Natural Fruit – Session 7.
	Tangerine	Natural Fruit – Session 8.
	Mango	Natural Fruit – Session 9.
	Lemon	Natural Fruit – Session 10.
	Guava and kiwi	Natural Fruit – Session 11.

Source: Authors.

Strategy 1: Through video, illustration, and oral presentation, the olfactory function and etiological factors of olfactory disorders were explained. The MedSmell® kit for daily home-based olfactory training was provided, together with detailed instructions for its use. Home olfactory training and nasal hygiene (nasal irrigation with 0.9% sodium chloride saline solution, using a 20mL needleless syringe and applying the solution to each nostril to the maximum extent allowed by the syringe) were

performed daily (at least three times per day). Training and hygiene monitoring sheets were completed for documentation purposes.

Strategies 2-11: Odors were presented approximately 2 cm from the patient's nose, first to one nostril (with the contralateral nostril occluded using the index finger), then to the other, and finally in both nostrils simultaneously. Initially, stimuli were provided with a visual cue and, if possible, with an associated gustatory cue, followed by presentation without a visual cue. A 30-second interval was maintained between stimuli. The Nasal Airflow-Inducing Maneuver (NAIM) technique was taught and performed during the sessions (characterized as a "prolonged yawn" with simultaneous retraction movement of the mandible, floor of the mouth, tongue base, and palate muscles, always with the lips closed) to stimulate retronasal olfaction. In addition to the kit, during each session patients were instructed to practice with the stimuli addressed in that session (a set of four primary odors). During the stimulus application, the patient was asked to establish an association (whenever possible), with an affective memory (e. g., apple and cinnamon cake baked by a family member at gatherings, or a moment when they received a bouquet). The outcomes of correct and incorrect responses were documented on separate forms.

4) Reassess olfactory function.

Number of sessions: 01.

Content: Knowledge of changes in the patient's history and clinical profile regarding orofacial motor skills.

Resources: Connecticut test kit – MedSmell®.

Strategy: Oral interview conducted using the same protocols applied at the beginning of the study. Accordingly, tests were reapplied to assess olfaction with the aforementioned instrument. It should be emphasized that neither the initial

assessment nor the reassessment was performed by the therapist, in order to minimize analysis bias.

Material Suplementar

Programa Fonoaudiológico de Reabilitação do Olfato (PROL)

I. Classificação Estatística Internacional de Doenças e Problemas Relacionados à Saúde (CID 11)

R43 Distúrbios do olfato e do paladar.

R43.0 Anosmia

R43.1 Parosmia

R43.2 Parageusia

R43.8 Outros distúrbios do olfato e do paladar e os não especificados

II. Principais achados

1) Recuperação incompleta e tardia da perda súbita do olfato por infecções virais, no caso, a COVID-19[13].

2) Alteração do paladar (disgeusia), do olfato (hiposmia ou anosmia), xerostomia e hipossalivação após o tratamento para COVID-19[12, 14].

3) Maiores riscos de percepção distorcida de odores (parosmia ou troposmia) em jovens acometidos por infecção viral (no caso: COVID-19), com prejuízos na qualidade de vida[15].

III. Objetivo Geral

1) Adequar a função olfativa

***IV. Objetivos Específicos e Respectivas Intervenções Terapêuticas*.**

1) Realizar entrevista e avaliação do olfato do paciente.

N.º de sessões: 01.

Recursos: Protocolo de anamnese, kit do teste de *Connecticut*.

Estratégias: Anamnese com dados de identificação, história pregressa da queixa, levantamento dos possíveis fatores etiológicos, tratamentos anteriores e atuais, impactos da hiposmia/anosmia nas atividades de vida diária e avaliação do olfato.

2) Respirar de modo nasal.

N.º de Sessões: 11.

Recursos: Espelho, figuras, materiais de sopro (estojo respiratório da Pró-Fono®, língua de sogra e bexiga), seringa para higienização com soro fisiológico e raspador de língua.

Estratégia 1: Por meio de vídeo, ilustração e exposição oral do terapeuta, será explanada sobre a função respiratória e demonstração sobre a higienização nasal e da língua, que deve ser diária (mínimo três vezes por dia).

Estratégias 2-11: Técnicas respiratórias incluindo inspiração nasal (I), pausa (P) e expiração nasal (E), com aumento gradativo do tempo, associadas à aplicação prévia de bandagem elástica em “v” invertido de glabela à asa do nariz (colocada no início e retirada no final da sessão pelo terapeuta), desde que não haja indicativos de alergia ao material. Os materiais de sopro serão utilizados para variação das atividades no processo terapêutico.

3) Detectar, discriminar, nomear e memorizar odores.

N.º de Sessões: 11.

Recursos: Kit da *MedSmel®l* com quatro frascos âmbar com disco de papel aquarela embebidos em essências de diferentes grupos olfativos: limão, rosa, cravo e eucalipto (2 ml cada), além de frascos com odores primários contidos no quadro abaixo:

Quadro. Lista dos odores primários, estímulos utilizados nas terapias semanais presenciais e em que sessão foram utilizados.

**Odores primários**	**Estímulos**	**Formato – Sessão**
Floral	Talco	Pote MedSmell do talco Johnson® - Sessão 2.
	Jasmim	Sabonete em barra Jasmim (Granado®) – Sessão 3.
	Lírio	Sabonete em barra da marca Phebo® (Lírio) – Sessão 4.
	Rosa	Sabonete em barra Nativa Spa, da marca Boticário® (Rosa) – Sessões 5 e 6.
	Laranjeira	Sabonete em barra da marca Natura®, Linha Ekos (Laranja e Flor de Laranjeira) – Sessão 7.
	Lavanda	Sabonete em barra da marca Natura®, Linha Ekos (Lavanda) – Sessão 8.
	Rosa	Rosa natural – Sessão 9.
	Camomila	Caixinha de chá (marca Leão®, sabor camomila) – Sessão 10.
	Erva doce	Caixinha de chá da marca Leão® (Erva Doce) – Sessão 11.
Resinoso	Mentol	Pote do MedSmell® – Sessões 2 e 10.
	Eucalipto	Óleo de Eucalipto da marca Granado® – Sessões 3 e 7.
	Pasta de dente de menta	Tubo de pasta de dente da marca Close Up® (sabor Menta Refrescante) – Sessões 4 e 11.
	Baunilha	Pote de baunilha da marca Dr. Oetker® – Sessões 5 e 9.
	Madeira	Lascas (obtidas em marcenarias) – Sessão 6.
	Plástico	Garrafa de plástico com água dentro (a garrafa foi deixada no sol no período anterior à sua utilização) – Sessão 8.
Aromático	Cravo	Pacotinho de cravo em grão da marca Arisco® – Sessão 2.
	Canela	Pacotinho de Canela em Pau (Marca Arisco®) – Sessão 3.

	Capim Limão	Caixinha de chá de Capim Limão da marca Leão® – Sessão 4.
	Café solúvel e em pó	Potinho de café solúvel e em pó (ambos da Marca Pilão® tradicional)– Sessão 5.
	Chocolate em barra e em pó	Potinho de Nescau em pó (Nestlé®) – Sessão 6.
	Alho	Pote de alho triturado em conserva da marca Arisco® – Sessão 7.
	Cebola	O próprio caule (produto real) – Sessão 8.
	Orégano	Pacotinho de orégano natural seco da marca Kitano® – Sessão 9.
	Paçoca (amendoim)	Pacotinho de “paçoquita” da marca Santa Helena® – Sessão 10.
	Coentro	Maço de Coentro Fresco (produto real) – Sessão 11.
	Cominho	Pacotinho de cominho em pó da marca Kitano® – Sessão 11.
Frutado	Laranja	Fruta natural e pote essência de laranja da marca Arcolor® – Sessão 2.
	Banana	Fruta natural – Sessão 3.
	Maracujá	Fruta natural e pote essência de maracujá da marca Arcolor® – Sessão 4.
	Abacaxi	Bolo e pote essência de abacaxi da marca Arcolor® – Sessão 5.
	Uva e morango	Fruta natural (uva) e pote essência de morango da marca Arcolor® – Sessão 6.
	Maçã	Fruta natural – Sessão 7.
	Tangerina	Fruta natural – Sessão 8.
	Manga	Fruta natural – Sessão 9.
	Limão	Fruta natural – Sessão 10.
	Goiaba e kiwi	Fruta natural – Sessão 11.

Fonte: Próprios autores.

Estratégia 1: Por meio de vídeo, ilustração e exposição, foi explanada sobre a função olfativa e os fatores etiológicos dos distúrbios do olfato. Foi entregue o *kit* da MedSmell® para treino olfativo diário no domicílio e orientado sobre como utilizá-lo. O

treinamento olfativo domiciliar e as higienizações nasais (lavagem com soro fisiológico 0,9% cloreto de sódio, seringa sem agulha de 20ml e aplicação do soro em cada narina na quantidade máxima da seringa) foram diárias (mínimo três vezes por dia) e preenchidos os controles do treinamento e higienizações em uma folha para registro.

Estratégias 2-11: Os odores foram disponibilizados a 2 cm do nariz do paciente, disposto em uma narina (com a oposta tampada com o dedo indicador), depois na outra e, finalmente, em ambas. Primeiro com pista visual e, se possível, com pista gustativa associada e, posteriormente, sem pista visual. Cabe ressaltar que foi dado intervalo entre os estímulos de 30 segundos. A técnica *Nasal Airflow-Inducing Maneuver* (NAIM) foi ensinada e realizada nas sessões ("bocejo" prolongado com movimento simultâneo de retração da mandíbula, do assoalho da boca, da língua, da base da língua e do palato muscular, sempre com os lábios fechados) para estimular o olfato retronasal. Além do *kit*, a cada sessão os pacientes eram orientados a treinar com os estímulos trabalhados na sessão (grupo de quatro odores primários). Durante a aplicação dos estímulos, foi solicitado ao paciente que fizesse a associação (sempre que possível) com alguma memória afetiva (exemplo: bolo de maçã com canela que alguém da família assava em encontros familiares ou um momento em que recebeu um ramalhete). Os resultados de acertos e erros foram transpostos em folha própria.

4) Reavaliar o olfato.

N.º de sessões: 01.

Conteúdo: Conhecimento de mudanças no histórico da paciente e do quadro clínico em motricidade orofacial.

Recursos: *kit* do teste de Connecticut – MedSmell®.

Estratégia: Entrevista oral com os mesmos protocolos do início da pesquisa. Assim, testes foram reaplicados para avaliar o olfato, com o uso instrumental supracitado.

Cabe ressaltar que tanto a avaliação quanto a reavaliação não foram realizadas pelo terapeuta, a fim de evitar viés de análise.
