# Supplementary material for: Speech therapy program for the rehabilitation of smell
Source: Codas. 2026 Mar 27;38(2):e20250086. doi: 10.1590/2317-1782/e20250086en (PMC13075829; doi:10.1590/2317-1782/e20250086en)
Supplement: Translation

# Translation: Programa fonoaudiológico para a reabilitação do olfato

**DOI:** 10.1590/2317-1782/e20250086pt

**Authors:** Carla Patrícia Hernandez Alves Ribeiro César, Keyllam Machado de Lima, Otávio Messias Silva de Oliveira, Lorena Mikaelly Nascimento Santos, Vanessa Silva Dantas, Danielle Ramos Domenis, Rafael Ciro Marques Cavalcante, Raphaela Barroso Guedes-Granzotti, Kelly da Silva

**Affiliations:** 1 Departamento de Fonoaudiologia, Universidade Federal de Sergipe – UFS - São Cristóvão (SE), Brasil.; 2 Programa Associado de Pós-graduação em Fonoaudiologia, Universidade Federal da Paraíba, Universidade Federal do Rio Grande do Norte e Universidade Estadual de Ciências da Saúde de Alagoas, Brasil.; 3 Departamento de Fonoaudiologia, Universidade Federal de Sergipe – UFS - Lagarto (SE), Brasil.; 4 Departamento de Farmácia, Universidade Federal de Sergipe – UFS - Lagarto (SE), Brasil.

**Keywords:** Transtornos do Olfato, Treinamento Olfativo, Olfato, Reabilitação, Fonoaudiologia

## Abstract

**Objetivo:**

Avaliar a eficácia da intervenção terapêutica fonoaudiológica voltada para as disfunções olfativas.

**Método:**

O programa fonoaudiológico de reabilitação olfativa foi aplicado em oito pacientes com queixa de diminuição olfativa por infecção viral persistente (1 a 2 anos pós-infecção) e presença de hiposmia pelo teste de Connecticut. A proposta consistiu em 13 sessões presenciais de 45 minutos (a primeira e a última de avaliação), em que foram realizadas estratégias envolvendo a conscientização do olfato; da respiração nasal e da necessidade de higienização frequente (nariz e cavidade intraoral); da percepção, da identificação e da contextualização dos estímulos odoríferos distribuídos por quatro odores primários; pela manobra Nasal Airflow-Inducing Maneuver; bem como associação com o paladar e treino domiciliar com o kit de treinamento olfativo da MedSmell®, por três vezes ao dia. O projeto foi previamente aprovado pelo comitê de ética e pelo Registro Brasileiro de Ensaios Clínicos.

**Resultados:**

Os pacientes apresentaram idade média de 53,29 anos±21,92, distribuídos igualmente entre os gêneros. Houve melhoras do olfato em todos os pacientes, sendo que em seis (75%) houve reversão para normosmia, um (12,5%) passou de anosmia para hiposmia moderada e outro (12,5%) de anosmia para hiposmia leve. O kit de treinamento olfativo foi utilizado no domicílio diariamente e os pacientes finalizaram o processo reconhecendo os efeitos benéficos da terapia.

**Conclusão:**

Houve eficácia da intervenção terapêutica fonoaudiológica voltada para a reabilitação dos distúrbios olfativos de ordem viral, havendo a necessidade de aumento da amostra para a generalização dos resultados.

## INTRODUÇÃO

O olfato é uma das funções mais importantes e antigas na escala da evolução humana, por ser por meio dele que é possível a percepção de diferentes estímulos odoríferos pelos receptores sensoriais presentes nas narinas. Essas estruturas possuem conexão sináptica direta com o bulbo olfatório na base do cérebro e este se conecta com múltiplas regiões corticais, bem como com o sistema límbico, permitindo seu envolvimento com os componentes afetivos dos odores, como o prazer e a aversão^(1)^.

Disfunções nessa percepção sensorial impactam significativamente a vida de uma pessoa, e podem ser decorrentes de diferentes fatores etiológicos como as infecções virais, os traumatismos cranioencefálicos, hábitos como o tabagismo, doenças neurodegenerativas e sistêmicas, bem como fatores iatrogênicos. Sendo que as disfunções olfativas ganharam grande destaque após a pandemia de COVID-19, por ser um sintoma notável em pacientes infectados e podendo estar presente até mesmo três anos após a infecção^(2)^.

Os pilares diagnósticos das disfunções olfativas são constituídos pela história clínica e pela aplicação de testes psicofísicos padronizados e validados para tal fim, que verificam desde a ausência do olfato (anosmia) até sua diminuição (hiposmia). Sendo assim, os testes mais utilizados e aceitos internacionalmente são o teste de Identificação do Olfato da Universidade da Pensilvânia (UPSIT - *University of Pennsylvania Smell Identification Test*)^(3)^ validado para a língua portuguesa^(4)^, o teste de *Connecticut* desenvolvido por pesquisadores americanos^(5)^, em 1988, e adaptado para o Brasil^(6)^, bem como o Sniffin’ Sticks^(7)^, já adaptado transculturalmente para a população pediátrica brasileira^(8)^, entre outros.

As intervenções terapêuticas são orientadas pelos seus fatores etiológicos, sendo que o treinamento olfativo é uma das possibilidades de tratamento. Quanto menor o grau da disfunção e a idade do paciente, melhores são os prognósticos, além da possibilidade do enfrentamento do fator etiológico, podendo haver limitações terapêuticas nesse sentido^(9)^.

As especialidades da Fonoaudiologia que podem colaborar no tratamento funcional do olfato são a Motricidade Orofacial e a Disfagia, uma vez que as áreas lidam diretamente com as regiões orofacial e cervical; e as disfunções do olfato e do paladar podem comprometer a qualidade de vida dos sujeitos acometidos^(10)^.

Justifica-se a atual pesquisa, pois, até o momento, não há publicações científicas de intervenção fonoaudiológica padronizada para pacientes com hiposmias de diferentes ordens etiológicas como as infecções virais (em especial ao SARS-CoV-2, desde 2020), aos traumatismos cranioencefálicos, às doenças neurodegenerativas como o Alzheimer e o Parkinson, ao próprio envelhecimento, como sequela de radiação e/ou quimioterapia do tratamento para o câncer craniocervical, entre outros.

Diante do exposto, a presente pesquisa teve por objetivo avaliar a eficácia da intervenção terapêutica fonoaudiológica voltada para as disfunções olfativas de pacientes com síndrome pós-Covid 19, por meio da comparação pré e pós-intervenção fonoaudiológica da avaliação do olfato por teste reconhecido nacional e internacionalmente.

## MÉTODO

Trata-se de um ensaio clínico randomizado, antes/depois, prospectivo e triplo-cego, devidamente aprovado pelo Comitê de Ética em Pesquisa (CAAE N.° 61448422.7.0000.0217 e Parecer N.° 5.658.792), assim como pelo Registro Brasileiro de Ensaios Clínicos (ReBEC) sob o número RBR-23k6dgq.

Para compor a amostra, foi realizado um levantamento de todos os prontuários de pacientes internados com COVID-19 entre 11 de março de 2020 e 22 de maio de 2022 em dois hospitais que foram referência para esses casos no estado de Sergipe. Em dezembro de 2023, foi realizado o contato telefônico para convidá-los a participar da pesquisa e, para os que aceitaram, foi agendada para assinatura do Termo de Consentimento Livre Esclarecido, entrevista e avaliação do olfato em dias e horários pré-estabelecidos, entre janeiro e fevereiro de 2024. De forma randomizada, dos 49 pacientes avaliados, 18 apresentaram queixa de disfunção olfativa persistente entre um e dois anos pós-infecção e treze ratificaram a hiposmia, sendo que esses treze foram convidados a participar do Programa Fonoaudiológico de Reabilitação do Olfato (PROL).

Cabe ressaltar que foi realizado cálculo amostral prévio, sendo considerado um tamanho de efeito grande (d = 0,8), um nível de significância de 5% (α = 0,05) e um poder estatístico de 80%, o número mínimo de sujeitos necessários para detectar uma diferença significativa entre as medições antes e depois da intervenção seria de 13 indivíduos. No entanto, devido aos critérios de elegibilidade e à não aceitação na participação da pesquisa, o estudo foi iniciado com nove e finalizado com oito pacientes, como pode ser constatado na seção de resultados.

Os critérios de inclusão foram pessoas que apresentaram Covid-19 na forma grave, com idade entre 18 e 60 anos, que permaneceram hospitalizadas para o tratamento da infecção viral respiratória, bem como aceitaram participar da fonoterapia. Os de exclusão foram pessoas com deficiências cognitivas, que testaram positivo para Covid-19 no momento das avaliações, que apresentaram dificuldades em responder oralmente os questionários e cumprir com as orientações terapêuticas e aqueles com uso medicamentoso que interferisse na avaliação e na reabilitação do olfato, como a carboplatina, a cisplatina, a ciclofosfamida, a doxorrubicina, o fluorouracil, o metotrexato, a levamisole e a vincristina, quer seja por inflamação/irritação da mucosa ou por neurotoxicidade. Pacientes que apresentaram queixas de alteração do olfato, mas obtiveram resultados dentro da normalidade também foram excluídos, em virtude de possível parosmia, disfunção sutil ou intermitente, hiperosmia, alterações específicas para certos odores, por disfunção apenas da função olfativa retronasal entre outros. Para a aplicação de tais critérios, os pacientes participaram de entrevista no dia da pesquisa, antes da avaliação propriamente dita.

Para evitar risco de viés^(11)^, foram adotadas as seguintes medidas: (1) aplicação de alocação dos pacientes por examinador, ou seja, os avaliadores não aplicaram a terapia e vice-versa. Para tanto, um dos coordenadores do projeto foi a única pessoa que teve os resultados das avaliações e das terapias realizadas, sendo instaurada uma central de resultados da pesquisa; (2) os recipientes para o teste de olfato foram padronizados e uniformes, a fim de que os pacientes não identificassem os estímulos-teste; (3) houve cegamento de pacientes, avaliadores de desfecho e profissionais (triplo cego), uma vez que os protocolos dos exames preenchidos, após realizados pelos avaliadores, foram acondicionados em envelopes pardos, selados e numerados pela central de resultados, a fim de que os avaliadores e terapeutas não pudessem ter acesso às informações; (4) todos os procedimentos adotados foram idênticos entre os pacientes^(12)^.

Em relação aos procedimentos, antes da avaliação e da reavaliação do olfato, foi realizado o teste de antígenos para a COVID-19 e o resultado foi fornecido em: detectado (positivo) ou não detectado (negativo). Aqueles que apresentaram resultado negativo foram encaminhados para a próxima etapa.

Para a avaliação do olfato, foi utilizado o Teste de *Connecticut* (*Connecticut Chemosensory Clinical Research Center*–CCCRC *test*) comercializado pela MedSmell®, padronizado e adaptado para o Brasil^(6)^. O *kit* utilizado para a pesquisa é composto por oito frascos contendo soluções de álcool n-butílico (butanol) em diferentes concentrações (4%, 1%, 0,4%, 0,1%, 0,05%, 0,01%, 0,005%) e um frasco com água destilada (controle), para a obtenção do limiar olfativo; oito frascos contendo substâncias odoríferas como café em pó, canela em pó, pó de talco, paçoca, chocolate em pó, sabonete neutro, naftalina – para a nomeação de tais estímulos e o oitavo frasco contendo o odor de mentol para a avaliação da aferência do nervo trigêmeo; uma máscara tapa olhos, um manual de instruções e folhas/tabelas para o registro das respostas.

A análise é quantitativa, identificando o limiar olfativo, e qualitativa pelo reconhecimento/nomeação dos estímulos odoríferos-teste. As narinas são testadas separadamente e coloca-se uma venda nos olhos do paciente para que estes permaneçam fechados. O resultado é a somatória da pontuação, obtendo-se a média aritmética conforme descrito abaixo.


$$\frac{\begin{array}{c} \text{limiar da narina D}+\text{limiar da narina E}+\;\\ \text{reconhecimento de odores da narina D}+\\ \text{reconhecimento de odores da narina E} \end{array}}{4}$$

(legendas: D = direita e E = esquerda)

A partir do resultado encontrado, faz-se a classificação de normosmia pela pontuação entre 6,0–7,0 pontos, de hiposmia entre 5,0–2,0 pontos (sendo leve entre 5,75–5,0 pontos, moderada entre 4,75–4,0 pontos e severa entre 3,75–2,0 pontos) e anosmia entre 1,75 e zero pontos.

O PROL foi constituído de 13 sessões semanais presenciais de 45 minutos cada, sendo as sessões um e treze de avaliação e reavaliação, respectivamente. Tendo como objetivo geral adequar a função olfativa e objetivos específicos, 1) Respirar nasalmente; 2) Detectar, discriminar, nomear e memorizar odores (sempre com higienização nasal prévia, uso de bandagem na sessão, realização da *Nasal Airflow Inducing Maneuver –* que é a “técnica do bocejo educado” com inspiração e expiração nasal profundas, com abaixamento mandibular sem a abertura dos lábios, gerando uma pressão intraoral e orofaríngea negativas permitindo, assim, estimular o olfato retronasal^(13)^, do paladar e da memória afetiva relacionada ao olfato, todos utilizados de forma conjunta com esse objetivo); e 3) Orientar acerca de atividades a serem realizadas no domicílio e da importância do olfato para a alimentação. Foi entregue o *kit* da MedSmell® para treino olfativo diário no domicílio e orientado sobre como utilizá-lo. Essa medida foi possível em virtude do financiamento da pesquisa pelo CNPq. O treinamento olfativo domiciliar e as higienizações foram diárias (mínimo três vezes por dia) e preenchidos os controles do treinamento em uma folha para registro. Cabe ressaltar que o PROL foi descrito a partir da seguinte estrutura: classificação estatística internacional de doenças e problemas relacionados à saúde (CID 11), principais achados^(12,14-16)^, objetivos (geral e específicos) com as respectivas intervenções fonoterapêuticas: número de sessões, conteúdo, recursos e estratégias (disponível no Material Suplementar).

Caso o paciente tivesse 15% de faltas, era desligado da pesquisa. Além disso, foi delineado o desfecho seletivo desejado, sendo classificado como resultado satisfatório os sucessos terapêuticos entre 80–100%, moderado entre 79–60%, regular entre 59–40%, fraco entre 39–20% e insatisfatório entre 19–0%.

## RESULTADOS

Dos oito pacientes, quatro eram do gênero feminino e quatro do masculino, com média de idade de 55,50 ± 21,24.

Os objetivos terapêuticos foram trabalhados sem alterações das estratégias delineadas previamente, sendo que os pacientes mostraram-se colaborativos e empenhados nas sessões, descrevendo melhoras progressivas do seu quadro clínico. Abaixo, os resultados comparativos dos momentos pré e pós-terapia com as respectivas condutas (Quadro 1).

**Quadro 1:** Resultados pré e pós-terapia olfativa, com as considerações dos resultados comparativos e suas respectivas condutas

**Sujeitos**	**Resultados pré-terapia (Pontuação média e classificação)**	**Resultados pós-terapia (Pontuação média e classificação)**	**Considerações dos Resultados Comparativos e Condutas**	**Sucesso Terapêutico**
1	3,5 pontos Hiposmia Severa	7,0 pontos Normosmia	Melhora da função olfativa, restabelecida integralmente. Conduta: Alta fonoaudiológica.	Satisfatório (100%)
2	4,0 pontos Hiposmia Moderada	6,0 pontos Normosmia	Melhora da função olfativa, restabelecida integralmente. Conduta: Alta fonoaudiológica.	Satisfatório (85,71%)
3	1,5 pontos Anosmia	4,0 pontos Hiposmia Moderada	Melhora da função olfativa, porém não houve restabelecimento total do olfato. Conduta: Continuidade da Terapia.	Regular (57,14%)
4	1,5 pontos Anosmia	5,25 Hiposmia Leve	Melhora da função olfativa, porém não houve restabelecimento total do olfato. Conduta: Continuidade da Terapia.	Moderado (75%)
5	3,25 pontos Hiposmia Severa	6,0 pontos Normosmia	Melhora da função olfativa, restabelecida integralmente. Conduta: Alta fonoaudiológica.	Satisfatório (85,71%)
6	3,75 pontos Hiposmia Severa	7,0 pontos Normosmia	Melhora da função olfativa, restabelecida integralmente. Conduta: Alta Fonoaudiológica.	Satisfatório (100%)
7	2,75 pontos Hiposmia Severa	7,0 pontos Normosmia	Melhora da função olfativa, restabelecida integralmente. Conduta: Alta Fonoaudiológica.	Satisfatório (100%)
8	4,5 pontos Hiposmia Moderada	7,0 pontos Normosmia	Melhora da função olfativa, restabelecida integralmente. Conduta: Alta Fonoaudiológica.	Satisfatório (100%)

**Fonte:** Próprios autores.

## DISCUSSÃO

O objetivo da presente pesquisa foi avaliar a eficácia da intervenção terapêutica fonoaudiológica voltada para as disfunções olfativas de pacientes com síndrome pós-Covid 19, uma vez que tais alterações impactam negativamente a saúde mental e a qualidade de vida dos sujeitos acometidos^(17)^. Muitos pacientes infectados pelo vírus SARS-CoV-2 apresentaram quadros agudos de perda do olfato, porém com recuperação da função. No entanto, entre 5% e 10% dos casos com anosmia apresentaram manutenção do quadro^(18)^. A literatura tem denominado tais manifestações como quadros de Covid longa, definida pela presença de sequelas multissistêmicas contínuas ou pós-agudas a longo prazo após o quadro de recuperação viral. Para a detecção precoce da Covid longa, inspeções auxiliares rotineiras (com exames de imagens e laboratoriais) são atualmente indispensáveis, incluindo os testes olfativos^(19)^.

Segundo a literatura os meios terapêuticos mais comumente utilizados para o tratamento das disfunções olfativas são os medicamentos tópicos, como os corticosteroides^(12)^, os suplementos com palmitoiletanolamida e luteolina^(20)^, aqueles que envolvem treinamento com estímulos olfativos, os mistos (uso medicamentoso e treinamento olfativo^(20,21)^ e a aplicação de fotobiomodulação de baixa frequência^(22)^. No presente estudo, a fim de se avaliar somente o impacto do treinamento olfativo, os pacientes que faziam uso medicamentoso foram excluídos da amostra para que esse fator não fosse um viés na análise. A fotobiomodulação também não foi utilizada pela necessidade de equipamentos de alto custo e profissionais habilitados para o seu uso.

Dentre as estratégias utilizadas no PROL, a combinação de estratégias que envolviam a lavagem nasal com solução salinizada, com o olfato retronasal, a bandagem (para dilatar as narinas durante as sessões presenciais), o trabalho concomitante com o paladar e as memórias afetivas envolvendo o olfato, revelaram resultados positivos na presente pesquisa.

Em relação à higienização nasal, justifica-se esse resultado pois, um nariz higienizado permite maior potência nasal e, consequentemente, facilita o modo respiratório nasal e limpeza. Ademais, contribui para uma melhor função do *clearance* mucociliar, uma vez que possibilita a eliminação de muco viscoso, a redução de mediadores inflamatórios e o aumento da frequência dos batimentos ciliares, apesar do desconhecimento, até o momento, de como isso ocorra^(22,23)^.

Salienta-se ainda que a manobra *Nasal Airflow-Inducing Maneuver (NAIM)* é utilizada com laringectomizados totais para estimular o olfato retroflexamente e assim permitir a percepção do odor e restaurar o olfato, independentemente do tempo decorrido da laringectomia^(24)^. No presente estudo, mostrou-se uma estratégia complementar exitosa, mesmo sem a laringectomia.

A estratégia principal do programa foi a oferta de diferentes estímulos odoríferos primários (floral, resinoso, aromático e frutado), conforme recomendado por revisão sistemática com metanálise^(25)^. Esse estudo revelou que o treinamento em pacientes infectados pelo vírus SARS-CoV-2, surtiu efeitos satisfatórios, de forma geral, sendo mais evidentes quando o quadro clínico era agudo.

Salienta-se ainda que, na literatura, são utilizados diferentes odores e tempos terapêuticos, sendo que a base do treinamento é o “prisma de odor” de Henning, em que os pacientes são orientados a treinarem no domicílio pela oferta padronizada de quatro odores (álcool fenil etílico (rosa), eucaliptol (eucalipto); citronelal (limão); eugenol (cravo) por duas vezes ao dia por 12 semanas^(2)^. Na presente pesquisa, o treinamento olfativo diário, no domicílio, foi realizado com o uso de estímulos odoríferos padronizados da MedSmell® e pelo oferecimento de onze sessões de terapia, com estímulos que pertenciam aos quatro odores primários, obtendo-se êxito na proposta. Os resultados obtidos na presente pesquisa ratificaram o exposto pela literatura. No entanto, além do treino domiciliar, foi feito acompanhamento presencial, associando-se o olfato com o paladar e revivendo experiências prévias com os estímulos trabalhados nas sessões. Isso ocorreu pelo fato de o olfato estar intimamente ligado ao sistema límbico. Assim, sempre que os estímulos olfativos eram trabalhados nas sessões, tentava-se recriar contextos significativos e discutir oralmente sobre as experiências afetivas dos pacientes com os odores apresentados, surtindo resultados suficientes nesse sentido.

A justificativa dessa melhora, de acordo com outra revisão sistemática que discute sobre o processo de regeneração do nervo olfatório^(26)^, sendo sua regeneração possível por diversos processos, como a renovação das células basais (tanto as horizontais quanto as globosas) localizadas no epitélio olfativo, por serem células precursoras dos neurônios receptores olfativos; pelo aumento da atividade enzimática das proteínas de ligação ao nucleotídeo guanina G(olf) subunidade alfa (GNAL) e adenilil ciclase (ADCY)-3 f, bem como pelo incremento da proteína ácida fibrilar glial (GFAP) e de neuroblastos migrantes. Desta forma, as mudanças bioquímicas e celulares do epitélio nasal parecem favorecer a um aumento do bulbo olfativo e, consequentemente, de diversas áreas centrais envolvidas no olfato, justificando o motivo do sucesso obtido pelo treinamento olfativo. Assim, hipotetiza-se que a estimulação repetida dos estímulos odoríferos promoveram neurogênese do bulbo olfatório e reforçaram as vias corticais olfativas.

Estudos que envolvam o treinamento olfativo consubstanciado com exames de imagens (tomografia computadorizada e ressonância magnética) são necessários para corroborar e favorecer a compreensão da neuroplasticidade ocorrida, similar ao estudo realizado com pesquisadores^(27)^ com pacientes com disfunção pós-traumática, elaborando escore prognóstico composto por cinco itens, com sensibilidade de 76,5% e especificidade de 97,1% para predizer se o sujeito terá ou não bons resultados ao treinamento olfativo.

Os resultados deste estudo apontaram implicações relevantes para a prática clínica fonoaudiológica, especialmente no contexto da reabilitação de disfunções olfativas de origem viral. A melhora significativa do olfato em todos os pacientes, com destaque para a reversão à normosmia em 75% dos casos, reforça o potencial terapêutico das estratégias fonoaudiológicas aplicadas, mesmo com uma amostra tida como reduzida. Tais achados revelam a importância da reabilitação olfativa como parte das abordagens clínicas em contextos pós-infecciosos, ampliando o escopo de atuação do fonoaudiólogo, podendo ser útil em quadros com outros fatores etiológicos que envolvam as anosmias/hiposmias. No âmbito da pesquisa, evidencia-se a necessidade de estudos com amostras maiores e delineamentos controlados, a fim de validar e consolidar a eficácia das intervenções. Do ponto de vista das políticas públicas, a inclusão de programas estruturados de reabilitação olfativa nos serviços de saúde pode contribuir para a melhoria da qualidade de vida de pacientes com sequelas virais, otimizando recursos e promovendo uma atenção mais integral à saúde sensorial dos sujeitos acometidos pela afecção tema deste estudo.

Em relação às limitações do presente estudo, o número da amostra reduzido pode ser elencado como uma delas, inviabilizando a generalização dos dados. Apesar do exposto, os benefícios observados e relatados pelos pacientes da pesquisa justificaram sua realização e divulgação de resultados.

Quanto às perspectivas futuras, sugere-se comparar os resultados com pacientes que tenham apresentado outros quadros clínicos que justifiquem a disfunção olfativa, bem como comparar o treinamento olfativo domiciliar exclusivo com o combinado (terapia e domiciliar), a fim de verificar se há diferenças entre essas modalidades. Outra possibilidade seria a aplicação de realidade virtual associada à oferta de estímulos odoríferos, aumentando a experiência do sujeito com a situação apresentada.

## CONCLUSÃO

A partir do programa de reabilitação fonoaudiológica do olfato, realizado sob a forma de ensaio clínico triplo cego, foi possível concluir que o referido programa destinado a pacientes com disfunção de ordem viral surtiu efeito satisfatório na maioria da amostra estudada, devendo os resultados serem interpretados com cautela uma vez que o tamanho da amostra foi reduzido.

## Supplementary Material

Supplementary material accompanies this paper.

Speech Therapy Olfactory Rehabilitation Program (PROL)

This material is available as part of the online article from https://doi.org/10.1590/2317-1782/e20250086en
